# Glucocorticoid-Induced Leucine Zipper: A Novel Anti-inflammatory Molecule

**DOI:** 10.3389/fphar.2019.00308

**Published:** 2019-03-27

**Authors:** Oxana Bereshchenko, Graziella Migliorati, Stefano Bruscoli, Carlo Riccardi

**Affiliations:** ^1^Department of Surgery and Biomedical Sciences, University of Perugia, Perugia, Italy; ^2^Section of Pharmacology, Department of Medicine, University of Perugia, Perugia, Italy

**Keywords:** glucocorticoid-induced leucine zipper, recombinant GILZ protein, GILZ, GILZ-derived peptide, anti-inflammatory molecules

## Abstract

Glucocorticoids (GCs) are the most commonly used drugs for treatment of autoimmune and inflammatory diseases. Their efficacy is due to their ability to bind cytoplasmic receptors (glucocorticoid receptors, GR) and other cytoplasmic proteins, thus regulating gene expression. Although GCs are potent life-saving drugs, their therapeutic effects are transitory and chronic use of GCs is accompanied by serious side effects. Therefore, new drugs are needed to replace GCs. We have identified a gene, glucocorticoid-induced leucine zipper (GILZ or tsc22d3), that is rapidly and invariably induced by GCs. Human GILZ is a 135-amino acid protein that mediates many GC effects, including inhibition of the NF-κB and MAPK pathways. Similar to GCs, GILZ exerts anti-inflammatory activity in experimental disease models, including inflammatory bowel diseases and arthritis. While transgenic mice that overexpress GILZ are more resistant, GILZ knockout mice develop worse inflammatory diseases. Moreover, the anti-inflammatory effect of GCs is attenuated in GILZ-deficient mice. Importantly, *in vivo* delivery of recombinant GILZ protein cured colitis and facilitated resolution of lipopolysaccharide-induced inflammation without apparent toxic effects. A synthetic GILZ-derived peptide, corresponding to the GILZ region that interacts with NF-κB, was able to suppress experimental autoimmune encephalomyelitis. Collectively, these findings indicate that GILZ is an anti-inflammatory molecule that may serve as the basis for designing new therapeutic approaches to inflammatory diseases.

## Introduction: Glucocorticoid-Induced Leucine Zipper (GILZ) Is a Glucocorticoid (GC)-Inducible Gene

Glucocorticoids (GCs) are important hormones able to regulate homeostasis of virtually all organs and tissues of the human body. GCs act mainly as regulators of transcriptional activity of a large number of genes, but also by control of epigenetic mechanisms (Cain and Cidlowski, 2017; Zannas and Chrousos, 2017). GCs have anti-inflammatory and immunosuppressive activities that involve nearly all arms of the inflammatory response. Accordingly, GCs are widely used for therapy. However, although GCs are potent anti-inflammatory drugs, their clinical effects are transitory and chronic use of GCs is accompanied by serious side effects, such as hypertension, hyperglycemia, osteoporosis, mood disorders and Cushing’s syndrome, that lead to discontinuation of therapy (Ayroldi et al., 2014). Therefore, new drugs that can substitute for GCs may provide a critical aid in suppressing inflammation. Most of the effects mediated by GCs, at physiological and pharmacological concentrations, are mediated by interactions with the glucocorticoid receptor (GR) (De Bosscher et al., 2003). GR is predominantly located in the cytoplasm as a multiprotein complex called the receptosome. After GC/GR interaction and consequent conformational changes, GR dissociates from the complex and translocates into the nucleus, where, as a dimer, it interacts with other transcription and coactivator factors and binds to specific DNA sequences, called glucocorticoid recognition elements (GREs). Almost all the effects of GCs, therapeutic and unwanted, are mediated by GR activity. Extensive efforts to separate beneficial from harmful gene activation by modulating GR activity have not yielded any success and GC toxicity is still a big issue in clinical practice. Since GC-induced effects are mostly due to the modulation of target gene expression, we initiated studies aimed at identifying proteins induced by GC treatment that mediate the anti-inflammatory effects, but not the side effects, of GCs. This is an emerging approach in drug design and is based on the development of peptide and mimetic drugs with decreased toxicity and enhanced specificity compared with conventional anti-inflammatory molecules.

Glucocorticoid-induced leucine zipper (GILZ, or tsc22d3) was discovered in our laboratory as a gene rapidly induced by dexamethasone (DEX), a synthetic GC (D’Adamio et al., 1997; Cannarile et al., 2001). *In silico* and mutational analyses of the GILZ promoter showed that at least three canonical GREs are present in the proximal region of the GILZ transcriptional start site (Asselin-Labat et al., 2004; Wang et al., 2004). More than 100 papers have been published showing that GILZ is one of the earliest transcriptional targets of GR, thus indicating that it is a good candidate as a downstream GC-induced effector molecule. GC signaling can also induce epigenetic modifications, such as changes in histone methylation and acetylation (Vockley et al., 2016). We have observed that GILZ binds and inhibits histone deacetylases in myoblasts, suggesting GILZ may also be involved in the effect of epigenetic changes induced by GCs (Bruscoli et al., 2010).

Of note, responsiveness of GC-inducible genes to activated GR may also be dependent on chromatin accessibility of the regulatory regions (enhancers and promoters) that may be influenced and affected by environmental stimuli. These epigenetic changes might influence expression of GC-induced genes, including GILZ (John et al., 2011; Stavreva et al., 2015). On the other hand, the secretion of GCs itself is a classic endocrine response to environmental exposures, including stress. Consequently, GILZ, induced by GCs, is produced in response to stressful events such as tissue damage, infection, anxiety or depression. Notably, it has been shown that major depressive disorder (MDD) patients with reduced GILZ expression in the hippocampus and amygdala showed a smaller hippocampal volume, suggesting the involvement of GILZ in stress-related disorders such as depression and anxiety (Frodl et al., 2012).

In this review, we describe experimental evidence suggesting that GILZ mediates the anti-inflammatory effects of GCs and that GILZ-based molecules have anti-inflammatory properties.

## GILZ Mediates the Anti-Inflammatory Activity of GCs

**FIGURE 1:**
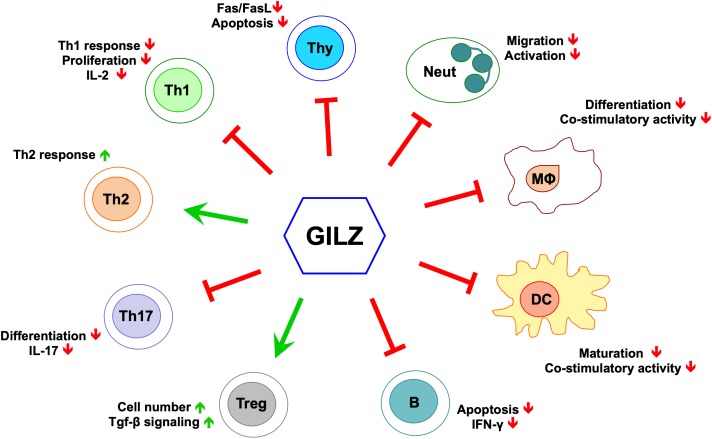
GILZ’s effects in cells of the immune system. Green arrows indicate positive regulation, red lines indicate negative regulation. Neut, neutrophils; Mϕ, monocytes/macrophages; DC, dendritic cells; B, B cells; Treg, T regulatory cells; Th17, T helper Type-17 cells; Th2, T helper Type-2 cells; Th1, T helper Type-1 cells; Thy, thymocytes.

In recent years, several laboratories including our own have produced a large amount of experimental evidence that supports the role of GILZ as a mediator of the anti-inflammatory activity of GCs (Figure 1) (Ayroldi et al., 2014; Ronchetti et al., 2015). GILZ is involved in the modulation of the same signaling immune responses and inflammation-related pathways implicated in GC-induced anti-inflammatory and immunosuppressive activities (Figure 1), suggesting that targeting GILZ can constitute a new approach to the treatment of inflammatory/autoimmune diseases.

**FIGURE 2:**
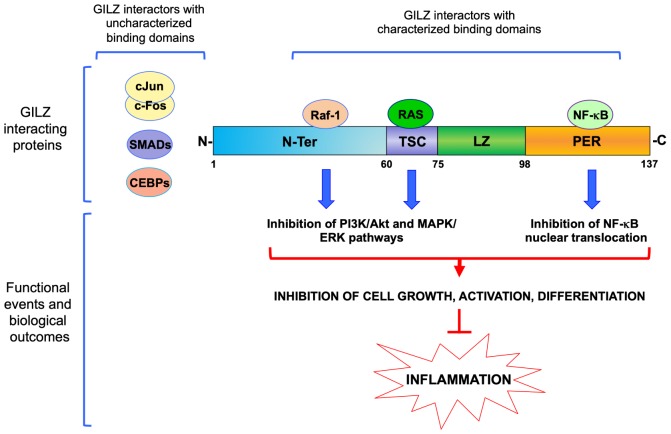
Molecular interactions of GILZ in the control of inflammation. Schematic representation of GILZ’s protein and its functional domains: N-Ter, N-terminal region (1–60 amino acids); TSC, TGF-β stimulated clone box (61–75 amino acids); LZ, leucine zipper domain (76–97 amino acids); PER, proline (P) and glutamic acid (E) rich region (98–137 amino acids). Proteins with characterized region of interaction are represented above GILZ protein diagram or aside, when specific regions of interaction have not been characterized yet. Raf-1, Raf-1 Proto-Oncogene, Serine/Threonine Kinase; RAS, Ras Proto-Oncogene, GTPase; NF-κB, nuclear factor kappa-light-chain-enhancer of activated B cells; cJun/cFos, proto-Oncogene components of an heterodimer also named Activator protein-1, AP-1; SMADs, Mothers against decapentaplegic homolog proteins; CEBPs, CCAAT-enhancer-binding proteins.

Glucocorticoid-induced leucine zipper was initially cloned in 1997 by comparing gene expression profiles of DEX-treated versus untreated murine T cells in differential display assays (D’Adamio et al., 1997). In this work, D’Adamio et al. (1997) showed that GILZ is involved in the regulation of T cell apoptosis. GILZ expression protects T cells from activated induced cell death through inhibition of Fas and FasL expression. The discovery of a human GILZ ortholog by Cannarile et al. (2001) indicated that GILZ is expressed and is induced by GCs in all peripheral blood cells, including T lymphocytes, monocytes and granulocytes. GILZ is also expressed in several other non-lymphoid tissues, and is ubiquitously upregulated by GCs (Cari et al., 2015). Much experimental evidence shows that GILZ interacts with several transcription factors and cellular signaling pathways (Table 1). Among those, GILZ physically interacts with essential factors in the control of inflammatory processes, including subunit p65 of nuclear factor kappa-light-chain-enhancer of activated B cells (NF-κB), c-Jun/c-Fos heterodimer (named Activator protein-1, AP-1), Raf-1, Ras, and CCAAT-enhancer-binding proteins (CEBPs) (Figure 2), as discussed in detail in the following sections, suggesting it has a remarkable central role in the control of inflammation.

**Table 1:** GILZ protein binding partners.

Gene name	Experimental system	Reference	PubMed ID
JUN	*In vitro* binding assay	Mittelstadt and Ashwell, 2001	11397794
FOS	*In vitro* binding assay	Mittelstadt and Ashwell, 2001	11397794
NFKB2 (p52)	Affinity Capture-Western	Ayroldi et al., 2001	11468175
NFKB1 (p65)	Affinity Capture-Western	Ayroldi et al., 2001	11468175
RAF1	Affinity Capture-Western	Ayroldi et al., 2002	12391160
RAS	Affinity Capture-Western	Ayroldi et al., 2007	17492054
SCNN1B	Affinity Capture-Western	Soundararajan et al., 2009	19380724
MYOD1	Affinity Capture-Western	Bruscoli et al., 2010	20124407
HDAC2	Affinity Capture-Western	Bruscoli et al., 2010	20124407
HDAC1	Affinity Capture-Western	Bruscoli et al., 2010	20124407
UBE2I	*In vitro* binding assay	Delfino et al., 2011	20671745
SUMO1	*In vitro* binding assay	Delfino et al., 2011	20671745
CASP8	Affinity Capture-Western	Delfino et al., 2011	20671745
SGK1	Affinity Capture-Western	Soundararajan et al., 2009	20947508
RAS	*In vitro* binding assay	Venanzi et al., 2014	24993177
TP53	*In vitro* binding assay	Ayroldi et al., 2014	25168242
TP53	Affinity Capture-Western	Ayroldi et al., 2014	25168242
MDM2	Affinity Capture-Western	Ayroldi et al., 2014	25168242
PU.1	Affinity Capture-Western	Ricci et al., 2017	28373208


## GILZ Interaction With NF-κB and MAPK/ERK Pathways in Inflammation

So far, the most relevant evidence of anti-inflammatory GILZ activities is related to interference with the NF-κB and Mitogen-Activated Protein Kinase (MAPK)/extracellular signal–regulated kinases (ERK) pathways. NF-κB is a transcription factor essential for the modulation of inflammatory and immune responses by controlling cytokine and chemokine production. Although NF-κB plays an essential, beneficial role in normal physiology, inappropriate regulation of NF-κB activity has been implicated in the pathogenesis of diseases including rheumatoid arthritis (RA), inflammatory bowel diseases (IBDs), osteoarthritis, atherosclerosis, asthma, multiple sclerosis (MS), and cancer (Barnes and Karin, 1997; Dai et al., 2009; Lawrence, 2009; Ben-Neriah and Karin, 2011).

Interference with NF-κB transcriptional activity by GCs in monocytes, macrophages, neutrophils, dendritic cells (DCs) and T lymphocytes is very important for the inhibition of the inflammatory response. We proposed that GILZ up-regulation induced by GCs is another way in which GCs control the activation of NF-κB. Ayroldi et al. (2001) demonstrated that GILZ directly binds to NF-κB and inhibits its activation (Figure 2). They showed that GILZ overexpression blocks nuclear translocation of the NF-κB/p65 subunit and it prevents the induction of pro-inflammatory NF-κB target genes in thymocytes, for example Interleukin-2 (IL-2) (Ayroldi et al., 2001; Di Marco et al., 2007). GILZ also mimics the effects of GCs on the differentiation of mature T cells, since GILZ overexpression in T cells of GILZ transgenic mice induces downregulation of the T helper (Th)-1 response and upregulation of the Th-2 response (Cannarile et al., 2006). The shift toward a Th2 response correlates with inhibition of NF-κB nuclear translocation in CD4+ T lymphocytes of intestinal lamina propria, and as a consequence, GILZ transgenic mice are less susceptible to Th1-mediated colitis.

Subsequent experimental evidence, obtained using several *in vitro* and *in vivo* models, further supported the link between anti-inflammatory activity of GILZ and inhibition of NF-κB. Berrebi et al. (2003) showed that GC inhibition of macrophage activation is at least in part achieved by GILZ-mediated inhibition of NF-κB. GILZ is constitutively expressed in mouse and human macrophages and in human monocytes, and is upregulated by GCs (Cannarile et al., 2001; Berrebi et al., 2003). In the THP-1 macrophage cell line, GILZ binds to the p65/NF-κB subunit and inhibits the expression of costimulatory molecules CD80 and CD86 and the production of chemokines Regulated on Activation, Normal T Cell Expressed and Secreted (RANTES) and Macrophage inflammatory protein 1 (MIP-1), thus mimicking the effect of GC treatment on macrophages (Berrebi et al., 2003). It has also been shown that GILZ mediates immunosuppressive effects in DCs. Overexpression of GILZ led to a tolerogenic effect of DCs in mediating T-cell activation by interfering with the NF-κB pathway (Cohen et al., 2006; Benkhoucha et al., 2014).

Beside the reported functions on cells of the immune system, GILZ has also been implicated in regulation of epithelial and endothelial inflammatory response. Indeed, GILZ silencing in human alveolar macrophages led to increased pro-inflammatory response and NF-κB activity (Hoppstadter et al., 2012). GILZ is also expressed in epithelial cells and GILZ peptide in human airway epithelial BEAS-2B cell line suppressed NF-κB activation (Eddleston et al., 2007). Furthermore, GILZ is expressed in synovial endothelial cells in RA, where it has been suggested to modulate inflammatory leukocyte recruitment via NF-κB. GILZ overexpression inhibits endothelial cell adhesive function through decreased expression of *E*-selectin, Intercellular Adhesion Molecule 1 (ICAM-1), C-C Motif Chemokine Ligand 2 (CCL2), C-X-C Motif Chemokine Ligand 8 (CXCL8), and Interleukin-6 (IL-6) and inhibition of Tumor Necrosis Factor (TNF)-stimulated leukocyte rolling, adhesion, and transmigration. Moreover, GILZ is downregulated in human umbilical vein endothelial cells by pro-inflammatory TNFα. Again, the anti-inflammatory effect of GILZ on endothelial cells involves the inhibition of NF-κB (Hahn et al., 2014). Moreover, GILZ-mediated inhibition of NF-κB nuclear translocation diminishes Cyclooxygenase 2 (COX-2) expression in bone marrow mesenchymal stem cells (MSCs) that were implicated in the pathogenesis and progression of RA (Chen and Tuan, 2008). GILZ expression was detected in the synovium of patients with RA and of mice with collagen-induced arthritis (CIA), and the severity of the disease was enhanced by GILZ silencing (Beaulieu et al., 2010; Ayroldi et al., 2014). The role of GILZ in the anti-inflammatory effect of MSCs in RA involves modulation of Th17 response toward a regulatory phenotype (Luz-Crawford et al., 2015). Another study confirmed that GILZ acts as a negative regulator of IL-17 production by T cells (Jones et al., 2015). GILZ expression in salivary gland is decreased in a mouse model of Sjögren’s syndrome, and GILZ overexpression inhibits production of IL-17 in salivary gland cells, suggesting that GILZ represents a potential target for diagnosis and treatment of Sjögren’s syndrome (Qin et al., 2015). Biological functions of Th17 cells are usually associated with pro-inflammatory activity implicated in the pathogenesis of autoimmune diseases. Yosef et al. (2013) proposed a dynamic regulatory network that comprises distinct Th17 cells with pro-inflammatory or regulatory activities. They indicated GILZ as a potential marker of Th17 cells with a non-pathogenic status (Yosef et al., 2013).

It has also been shown that GILZ dampens the pro-inflammatory effects of TNFα in human adipocytes by inhibiting p65/NF-κB nuclear translocation (Lee et al., 2016).

We have also shown that GILZ plays an important role in the homeostasis of B-cells (Bruscoli et al., 2015, 2018). GILZ deficiency leads to increased B-cell survival through elevated NF-κB activity and B-Cell CLL/Lymphoma 2 (Bcl-2) overexpression. B lymphocytosis observed in gilz deficient mice suggests that GILZ could be implicated in the pathogenesis of B-cell disorders such as hematological and/or autoimmune/inflammatory diseases. In this regard, a recent study reports a reduced GILZ expression in B cells of patients with systemic lupus erythematosus (SLE) (Jones et al., 2016).

The influence of GILZ on the expression of Bcl-2 family members is consistent with observations indicating that GILZ overexpression inhibits apoptosis in T lymphocytes through down-regulation of Bcl-2 Interacting Mediator Of Cell Death (Bim), a pro-apoptotic member of the Bcl-2 family (Asselin-Labat et al., 2004). GILZ also inhibits apoptosis in cardiomyocytes, where the pro-survival isoform of Bcl-XL protein is overexpressed when GILZ is overexpressed (Aguilar et al., 2014).

Interestingly, it has been shown that a GILZ-based peptide inhibits p65/NF-kB activation in lipopolysaccharide (LPS)-stimulated THP-1 myeloid cell line (Srinivasan et al., 2014). More recently, another study showed that a peptide corresponding to the 98–134 amino acids of GILZ inhibits LPS-induced p65/NF-kB nuclear translocation in retinal Müller cells (Gu et al., 2018a).

Collectively, these findings suggest that GILZ or GILZ-based peptides mimic the effect of GCs on NF-κB inhibition and GILZ expression is sufficient to achieve efficacious suppression of NF-κB-induced inflammation (Ngo et al., 2013; Ayroldi et al., 2014).

Many anti-inflammatory effects of GCs depend on cross-talk with the MAPK signaling pathway. We have shown that GILZ interacts with the MAPK pathway by binding to Raf-1 and Ras (Figure 2) (Ayroldi et al., 2002, 2007). GILZ interaction with activated Ras results in inhibition of both the Phosphoinositide 3-kinase (PI3K)/Akt and ERK pathways, thus dampening cell growth (including tumor cell growth) and differentiation. Also, GILZ mediates DEX-induced inhibition of the MAPK/ERK pathway in human airway epithelial cells (Liu et al., 2013). Specific genetic ablation of GILZ in myeloid cells resulted in an increased susceptibility to LPS-induced macrophage activation through hyperactivity of the MAPK/ERK pathway (Hoppstadter et al., 2015). Furthermore, in cooperation with Annexin-1, another mediator of anti-inflammatory effects of GCs, GILZ inhibits LPS-induced macrophage activation by enhancement of the ERK and JUN N-Terminal Kinase (JNK) MAPK pathways (Yang et al., 2009), and neutrophil migration into inflamed tissues (Ricci et al., 2017). In addition, GILZ also inhibits neutrophil activation in a mouse model of C. albicans infection (Ricci et al., 2019).

Inhibition of oncoprotein Ras affects both the MAPK and PI3k/Akt pathways that are involved in cell survival and activation of immune responses. Forkhead family (Fox) transcription factors are mediators in the PI3K/Akt pathway in the homeostasis of immune cells, including T lymphocytes (Peng, 2008). Among these factors, FoxO3 is phosphorylated and inactivated by activated Akt. It has been shown that GILZ inhibits FoxO3 activity and is part of a PI3K/Akt-FoxO3 signaling pathway that controls cell proliferation (Latre de Late et al., 2010).

A recent interesting report by Hoppstadter et al. (2016) showed that GILZ is induced in macrophages by curcumin, a natural product with anti-inflammatory properties. Hoppstadter et al. (2016) showed that curcumin inhibits inflammatory activities of the NF-κB and MAPK/Erk pathways and subsequent TNFα production via GILZ (Hoppstadter et al., 2016).

## GILZ and Other Inflammatory Signaling Pathways

GILZ is able to inhibit the function of AP-1, a transcription factor pivotal for activation of immune cells during inflammation (Schonthaler et al., 2011). GILZ heterodimerizes with components of AP-1, c-Fos and c-jun (Mittelstadt and Ashwell, 2001), and GILZ overexpression inhibits production of IL-2, a cytokine that plays a central role in T-cell homeostasis and activation (Ayroldi et al., 2001; Mittelstadt and Ashwell, 2001; Di Marco et al., 2007). T-cell activation, also through IL-2 production, suppresses GILZ expression (D’Adamio et al., 1997; Ayroldi et al., 2001; Asselin-Labat et al., 2004). This reciprocal inhibitory activity between T-cell activation and GILZ expression indicates that GILZ is important to maintain T-cell anergy, thus suggesting that modifications of GILZ expression and/or functions may have relevant effects in the control of inflammatory processes. Recently, we demonstrated that in GILZ-deficient mice, interferon-γ (IFNγ) production by B cells is increased, depending at least in part on enhanced AP-1 transcriptional activity. GILZ binds AP-1, inhibits its nuclear translocation and hence dampens AP-1-mediated transcriptional activation of IFNγ promoter, thus regulating B cell activities and the development of inflammatory diseases (Bruscoli et al., 2018).

As already mentioned, GILZ, like GCs, supports T cell differentiation toward the Th2 phenotype (Cannarile et al., 2006). An appropriate balance between inflammatory and anti-inflammatory signals is dependent on the regulated activity of different T cell subpopulations and the ability of tissue to regulate the immune response (Hall et al., 2011). In particular, T regulatory (Treg) cells participate in modulation of inflammatory response and their deregulation has been shown to be involved in many inflammatory diseases (Rudensky, 2011). It is now clear that Treg induction is important for GC-induced therapeutic effects. GCs are able to synergize with Transforming growth factor beta (TGF-β) in regulating the expression of Foxp-3, a transcription factor essential for Treg generation (Rudensky, 2011; Bereshchenko et al., 2014). Foxp-3 expression is in fact induced by TGF-β pathway activation that is due to Mothers against decapentaplegic homolog (SMAD) protein phosphorylation; interaction of TGF-β with specific receptors induces SMAD phosphorylation and consequently increases Foxp-3 expression. Bereshchenko et al. (2014) showed that GILZ is necessary for GC-mediated Treg cell induction. This effect is due to GILZ interaction with SMAD proteins, thus synergizing with the TGF-β activity in enhancing FoxP3 expression (Bereshchenko et al., 2014). Notably, the therapeutic effect of GCs was lacking in a colitis model in GILZ knockout mice (Bereshchenko et al., 2014).

CEBPs are leucine zipper (LZ) transcription factors involved in diverse cellular responses including cell proliferation and differentiation, and also in inflammatory processes. There are several studies indicating a role for CEBP members in mediating GC effects (Roos and Nord, 2012). CEBPs are transcription factors that hetero-dimerize with many other proteins, including GC-target proteins such as MAPK phosphatase 1(MKP1)/dual specificity phosphatase 1 (DUSP1) (Kassel et al., 2001).

It has been shown that GILZ binds CEBPδ and inhibits peroxisome proliferator-activator receptor (PPAR)-gamma 2 expression, thus resulting in inhibition of differentiation adipogenesis in MSCs (Shi et al., 2003). Moreover, GILZ overexpression inhibits CEBPα expression and function, thus promoting osteogenesis in MSCs (Zhang et al., 2008). Although there is no direct evidence that GILZ is involved in the control of immunoregulatory molecules produced by MSCs, as GCs do, the aforementioned evidence supports the idea that GCs also affect CEBP activity through GILZ expression. Thus, more studies are warranted to understand the interplay between GILZ and CEPBs in mediating the anti-inflammatory effects of GCs in MSCs.

Serum/Glucocorticoid Regulated Kinase 1 (SGK1) is another important kinase that participates in the control of inflammation. Aberrant SGK1 expression is observed in a wide variety of diseases, including lung fibrosis, diabetic nephropathy, glomerulonephritis, obstructive nephropathy, liver cirrhosis, pancreatitis, and Crohn’s disease (Artunc and Lang, 2014). Pharmacologic inhibition of SGK1 or suppression by small interfering RNA enhances pro-inflammatory cytokine production (including TNF, IL-12, and IL-6) in Toll Like Receptor (TLR)-activated monocytes. Lack of SGK1 results in increased phosphorylation of inhibitor of NF-kB kinase (IKK) and inhibitor of NF-kB Alpha (IκBα), and consequent increased NF-κB/p65 transcriptional activity in LPS-stimulated cells (Zhou et al., 2015). Interestingly, both SGK-1 and GILZ are up-regulated by aldosterone in the kidney and regulate the activity of Epithelial Na(+) Channel (ENaC), a sodium transport channel (Bhalla et al., 2006). More recently, it has been reported that reduced SGK-1 and GILZ expression was concomitant with increased IL-6 expression in patients with MDD (Frodl et al., 2012). These indications suggest a possible cooperation of SGK-1 and GILZ in the control of inflammatory response pathways.

Many members of the nuclear receptor superfamily are involved in the modulation of inflammation. In particular, the PPARs, like GR, have anti-inflammatory properties because they reduce expression of several pro-inflammatory cytokines, chemokines and cell adhesion molecules. Three isoforms, PPAR-α, PPAR-β/δ, and PPAR-γ, form heterodimers with retinoic acid receptors. Deficiency of PPAR-α in mice impairs DEX-induced inhibition of NF-κB, TNF-α production, cell migration, and COX-2 production. Moreover, PPAR-α contributes to the transcriptional up-regulation of GILZ by GCs (Cuzzocrea et al., 2008). GILZ over-expression inhibits PPAR-γ2 expression and blocks adipocyte differentiation (Zhang et al., 2008). The interplay between PPARs, GCs and GILZ is still not well characterized, and further studies are warranted to clarify whether GILZ also affects PPAR functions in inflammatory processes.

Estrogen receptor (ER), another member of the nuclear receptor superfamily, regulates GILZ expression. Tynan et al. (2004) showed that estrogens modulate GILZ expression in MCF-7 human breast cancer cells. Again, crosstalk between the GR and ER nuclear receptor members in inflammation might be therapeutically relevant and anti-inflammatory activity of GILZ may play a relevant role in this interplay (Cuzzocrea et al., 2007).

Together, these studies indicate that GILZ acts as a factor necessary for the maintenance of quiescence of immune cells. GILZ is downregulated upon activation of many cell types, including T and B lymphocytes, macrophages and DCs (D’Adamio et al., 1997; Lebson et al., 2011; Bruscoli et al., 2018; Hoppstadter et al., 2019), and is required for their proper activation, maturation and differentiation.

## GILZ Structure-Based Design of Putative Anti-Inflammatory Molecules

The mouse GILZ gene encodes a 137-amino acid (aa) LZ protein (D’Adamio et al., 1997). Human GILZ is a 135-aa protein, almost identical to its murine homologue (97% identity) (Cannarile et al., 2001). GILZ is composed of three domains—a TGF-β-stimulated clone (TSC) box (aa positions 40–75), a central LZ domain (aa 76–97), and a C-terminal domain endowed with a proline and glutamic acid rich (PER) region (aa 98–137). We also characterized a transcriptional variant, named long (L)-GILZ, that shares with GILZ all the conserved domains including the TSC box, LZ, and PER region, but differs in the N-terminal domain, and is encoded by an upstream alternative exon 1 (Bruscoli et al., 2012). Unlike most LZ proteins, which are usually transcription factors, GILZ isoforms do not contain a basic-rich region, and so far there has been no formal demonstration of direct binding to DNA; GILZ is mostly located in the cytoplasm, although it may also be present in the nucleus (Shi et al., 2003).

Different domains have been identified as essential for direct protein–protein binding interactions with different molecules. For example, the TSC-box domain is important for binding with Ras, while Raf-1 binds the N-terminal portion of GILZ. Both Raf-1 and Ras are activators of MAPK/ERK and PI3K/Akt pathways and GILZ expression negatively modulates both pathways (Ayroldi et al., 2002, 2007; Asselin-Labat et al., 2004; Venanzi et al., 2014). Interestingly, both c-Fos and c-Jun, proteins containing LZ domains and components of the transcription factor AP-1, are efficiently retained by the GILZ N-terminal portion (aa 1–60), which lacks the LZ domain necessary for homo-dimerization, indicating that the LZ domain is not necessary for GILZ to bind AP-1 components (Di Marco et al., 2007). This evidence opens an interesting question about the role of the LZ domain in GILZ. Usually, a LZ region is a protein–protein interaction motif in which there is a leucine every seventh residue in an α-helix over a short stretch of protein, forming a stable coiled-coil structure. This motif is important for homo- and heterodimerization with a similar motif to promote specific DNA binding by a basic region adjacent to the LZ motif. The LZ model was originally proposed on the basis of the leucine distribution in LZ regions of proteins. It is now known to be common to over 40 proteins, including C/EBPs, Myc, Fos, Jun, and cAMP Responsive Element Binding Protein (CREB), involved in stress responses and the regulation of cell survival and proliferation (Jindrich and Degnan, 2016). LZ proteins can also associate with non-identical partners to form heterodimers composed of two different subunits, thus greatly expanding the repertoire of functions that these proteins can display. Although the LZ domain of GILZ is highly conserved, it has not yet been demonstrated to be necessary for hetero-dimerization with other LZ proteins. It is possible that the presence of the TSC-box domain, close to the LZ domain, limits the capacity of the GILZ LZ domain to bind other LZ family proteins. Interestingly, GILZ also binds to other LZ proteins such as CEBPα, CEBPβ and CEBPγ, but the region responsible for direct binding has not yet been identified (Shi et al., 2003; Pan et al., 2014).

Importantly, much experimental evidence reporting GILZ overexpression in mice, as well as *in vivo* delivery of recombinant GILZ protein, suggests that GILZ mimics some of the anti-inflammatory effects of GCs, including inhibition of T cell activation/differentiation and macrophage activation (D’Adamio et al., 1997; Ayroldi et al., 2001; Berrebi et al., 2003; Cannarile et al., 2009; Pinheiro et al., 2013; Bereshchenko et al., 2014; Hoppstadter et al., 2015), thus supporting the idea that GILZ protein may serve as a basis for rational drug design to achieve similar anti-inflammatory effects to GCs but with reduced side effects.

The first demonstration that recombinant GILZ protein has anti-inflammatory effects was by Cannarile et al. (2009), when a transactivator of transcription (TAT)–GILZ fusion protein, injected subcutaneously in a mouse model of Dinitrobenzene sulfonic acid (DNBS)-induced colitis, was found efficacious in ameliorating signs of colon inflammation (Cannarile et al., 2009). It has also been demonstrated that hydrodynamic delivery of GILZ-expressing vectors containing the TAT–GILZ sequence was sufficient to prevent LPS-induced lethal inflammation (shock) (Pinheiro et al., 2013). In addition, Vago et al. (2015) have shown that TAT–GILZ fusion protein efficiently induces a pro-apoptotic program *in vivo* that promotes resolution of neutrophilic inflammation induced by LPS. Recently, it was shown that *in vivo* administration of TAT–GILZ fusion protein in a mouse model of acute kidney injury exerts protective effects by promoting neutrophil and T cell polarization toward an anti-inflammatory phenotype (Baban et al., 2018).

The use of GILZ-derived peptides containing the critical aa 120–123 necessary for GILZ interaction with NF-κB (Di Marco et al., 2007) has been shown to effectively counteract neuroinflammation in a mouse model of MS (Srinivasan and Janardhanam, 2011). Recently, an *in vivo* injection of GILZ-based peptide prevented light-induced photoreceptor apoptosis and protected the retina from degeneration in Sprague Dawley rats, thus demonstrating the therapeutic efficacy of GILZ-based peptide in degenerative retinal diseases (Gu et al., 2018b).

The inhibitory effect on neuroinflammation was also reported in a study where transgenic mice overexpressing GILZ showed reduced damage and inflammation upon spinal cord injury (SCI) compared with wild-type controls (Esposito et al., 2012). Interestingly, a different study showed that, in the same SCI model, genetic ablation of GILZ in T lymphocytes led to reduced lesions and inflammation, suggesting that GILZ is involved in inflammatory processes upon SCI (Mazzon et al., 2014). This apparent contradiction is probably because, apart from its direct immunomodulatory role on T cells (D’Adamio et al., 1997; Ronchetti et al., 2015) GILZ is also expressed in neurons and may have functions in cells involved in neuronal damage (Srinivasan and Lahiri, 2017). Further studies are warranted to define the role of GILZ in the central nervous system.

Finally, forced GILZ overexpression in endothelial cells inhibits their activation and production of chemoattractant molecules, through NF-κB inhibition, thus reducing leukocyte recruitment to the site of inflammation (Cheng et al., 2013).

## Conclusion

Data indicate that GILZ protein and GILZ-based molecules have therapeutic efficacy and are non-toxic to cells and mice. Therefore, novel drugs in the area of “inflammatory/autoimmune” diseases, including chronic, acute and lethal inflammation (shock), can be developed based on GILZ and its molecular interactions. Moreover, those drugs will be of interest for many inflammation-based degenerative diseases and possibly cancer. In the near future, we propose to identify novel anti-inflammatory drugs designed on the basis of the GILZ protein structure and described molecular interactions of GILZ that have been characterized in recent years. The efficacy, toxicity and molecular effects of these drugs will be evaluated in cellular systems and in established *in vivo* models of IBD and RA. A combination of experimental and computational techniques, together with a deep knowledge of the determinants of protein–protein and protein–ligand interactions, is necessary for the successful design of small compounds based on GILZ that will be efficacious for the treatment of inflammatory and autoimmune diseases.

## Author Contributions

OB, GM, and CR conceived the manuscript. OB and SB wrote the manuscript. SB designed the table and the figures. All authors approved the final version of the manuscript.

## Conflict of Interest Statement

The authors declare that the research was conducted in the absence of any commercial or financial relationships that could be construed as a potential conflict of interest.
